# Readout-segmented multi-shot diffusion-weighted MRI of the knee joint in patients with juvenile idiopathic arthritis

**DOI:** 10.1186/s12969-017-0203-z

**Published:** 2017-10-12

**Authors:** Alexander Sauer, Mengxia Li, Annette Holl-Wieden, Thomas Pabst, Henning Neubauer

**Affiliations:** 1 Department of Diagnostic and Interventional Radiology, University Hospital Wuerzburg, 97080 Wuerzburg, Germany; 2 Department of Radiation Oncology, University Hospital Wuerzburg, 97080 Wuerzburg, Germany; 3 Department of Paediatrics, University Hospital Wuerzburg, 97080 Wuerzburg, Germany; 4 Department of Diagnostic and Interventional Radiology, University Hospital Ulm, Albert-Einstein-Allee 23, Ulm, 89081 Germany; 5 SRH Clinic of Radiology, 98527 Suhl, Germany

**Keywords:** Diffusion-weighted MRI, Juvenile idiopathic arthritis, Synovitis

## Abstract

**Background:**

Diffusion-weighted MRI has been proposed as a new technique for imaging synovitis without intravenous contrast application. We investigated diagnostic utility of multi-shot readout-segmented diffusion-weighted MRI (multi-shot DWI) for synovial imaging of the knee joint in patients with juvenile idiopathic arthritis (JIA).

**Methods:**

Thirty-two consecutive patients with confirmed or suspected JIA (21 girls, median age 13 years) underwent routine 1.5 T MRI with contrast-enhanced T1w imaging (contrast-enhanced MRI) and with multi-shot DWI (RESOLVE, b-values 0–50 and 800 s/mm^2^). Contrast-enhanced MRI, representing the diagnostic standard, and diffusion-weighted images at b = 800 s/mm^2^ were separately rated by three independent blinded readers at different levels of expertise for the presence and the degree of synovitis on a modified 5-item Likert scale along with the level of subjective diagnostic confidence.

**Results:**

Fourteen (44%) patients had active synovitis and joint effusion, nine (28%) patients showed mild synovial enhancement not qualifying for arthritis and another nine (28%) patients had no synovial signal alterations on contrast-enhanced imaging. Ratings by the 1st reader on contrast-enhanced MRI and on DWI showed substantial agreement (κ = 0.74). Inter-observer-agreement was high for diagnosing, or ruling out, active arthritis of the knee joint on contrast-enhanced MRI and on DWI, showing full agreement between 1st and 2nd reader and disagreement in one case (3%) between 1st and 3rd reader. In contrast, ratings in cases of absent vs. little synovial inflammation were markedly inconsistent on DWI. Diagnostic confidence was lower on DWI, compared to contrast-enhanced imaging.

**Conclusion:**

Multi-shot DWI of the knee joint is feasible in routine imaging and reliably diagnoses, or rules out, active arthritis of the knee joint in paediatric patients without the need of gadolinium-based i.v. contrast injection. Possibly due to “T2w shine-through” artifacts, DWI does not reliably differentiate non-inflamed joints from knee joints with mild synovial irritation.

## Background

Juvenile idiopathic arthritis (JIA) is the most common entity of chronic arthritidis in children and adolescents with an incidence of about 4 to 10 patients per 100,000 children [1]. In most instances, MR imaging is more sensitive than physical examination for detecting active joint inflammation [2]. MRI is also useful for excluding non-rheumatological disease, for assessing inflammatory disease activity and for monitoring response to anti-inflammatory therapy [3]. Routine MR imaging of synovitis presently depends on T1-weighted scans with intravenous application of gadolinium-based contrast material [4]. In addition to known issues of allergic reactions and of gadolinium contrast injection in patients with impaired renal function, recent reports on cerebral gadolinium deposition after contrast-enhanced MRI [5, 6] have raised new concerns about the long-term safety of gadolinium-containing contrast agents. So there is a need for new gadolinium-free MRI techniques, also in young patients with arthritis. The first report on diffusion-weighted imaging (DWI) of synovitis in children was published in 2012 [7]. Since then, several studies have confirmed the feasibility and the potential diagnostic value of this technique [8–10]. However, standard DWI with single-shot diffusion-weighted imaging and echo-planar readout (single-shot DWI), as used in stroke imaging since the early 1990s, suffers from several technical limitations resulting in frequent imaging artifacts and low spatial resolution. In effect, discrimination of inflamed synovium is impossible or lacks diagnostic confidence in a significant proportion of patients [10, 11]. Recently, a refined DWI technique, that is readout-segmented multi-shot echo-planar DWI (multi-shot DWI), has become available on clinical scanners and promises higher spatial resolution, better delineation of small anatomical structures and less artifacts from magnetic field inhomogeneities [12, 13] and from the presence of joint effusion.

We undertook our study to investigate image quality and diagnostic performance of multi-shot DWI for synovial imaging in JIA patients with arthritis of the knee joint.

## Methods

### Patient population

Our study group includes 32 consecutive patients, that is 21 girls and 11 boys, who had both multi-shot DWI and contrast-enhanced MR imaging of the knee for routine diagnostic work-up between January 2014 and July 2016. Median patient age was 13 years, ranging from 2 to 14 years. Another eight patients, who had multi-shot DWI within this period of time, but no contrast-enhanced sequences, were excluded from the analysis for a lack of reference standard. Eventually, we analysed 24 JIA patients, diagnosed according to the International League of Associations for Rheumatology (ILAR) criteria [14], and another eight patients with sub-acute or chronic arthralgia, in whom JIA was eventually ruled out and who served as negative controls in our study. Of the 24 JIA patients, 15 patients had previously been diagnosed with JIA and were referred to follow-up MRI, 11 of these patients with on-going anti-inflammatory treatment (oral non-steroidal *n* = 6, methotrexate *n* = 4, adalimumab *n* = 1) and four patients without on-going treatment. Nine patients were newly diagnosed with JIA, with three patients being on oral non-steroidal anti-inflammatory treatment at the time of the MRI examination.

All study work was conducted in accordance with the Helsinki Declaration. Informed written consent was obtained from the legal guardians of all patients for all diagnostic procedures. A waiver was granted by the Institutional Review Board for the retrospective analysis of imaging data.

### MRI examination

All patients underwent clinical routine MRI of the knee joint on the same 1.5 Tesla scanner (Magnetom Aera, Siemens Healthcare, Erlangen) in supine position with a cubital intravenous line in place. Standard sequences were first scanned as needed for clinical imaging (e.g. coronal T2w TIRM, sagittal PDw with fat saturation, T1w TSE). Diffusion-weighted scans were acquired prior to injection of contrast agent using the following typical parameters: transverse multi-shot DWI (RESOLVE, Siemens Healthcare, Erlangen, TR = 6600 ms, TE = 57 ms, 5 read-out segments, low b-value = 0 s/mm^2^ (*n* = 11) or 50 s/mm^2^ (*n* = 21) and high b-value = 800 s/mm^2^, 3-scan trace-weighted, parallel imaging GRAPPA iPAT = 2, monopolar gradient scheme, one average for low b-value, two averages for b = 800 s/mm^2^, epi factor 63, echo spacing 0.38 ms, 34 slices, voxel size 1.5 × 1.5 × 3 mm^3^, field of view 200 mm, acquisition time 6 min 18 s). Scanning parameters were adapted as clinically needed for optimal fit in some patients. After intravenous injection of one weight-adapted standard dose of gadoterate meglumine (Dotarem, Guerbet, Paris, France), we acquired contrast-enhanced MRI (transverse T1w TSE with Dixon fat saturation), followed by additional coronal or sagittal scans, if clinically necessary. All but two patients were examined feet-first and with a dedicated multi-channel knee coil. Two young patients, 2 and 3 years of age, had MRI of the lower extremities including both knee joints scanned in supine position head-first with phased-array body coils in place and with sedation administered by a paediatric anesthesiologist.

### Image processing and analysis

**Fig. 1:**
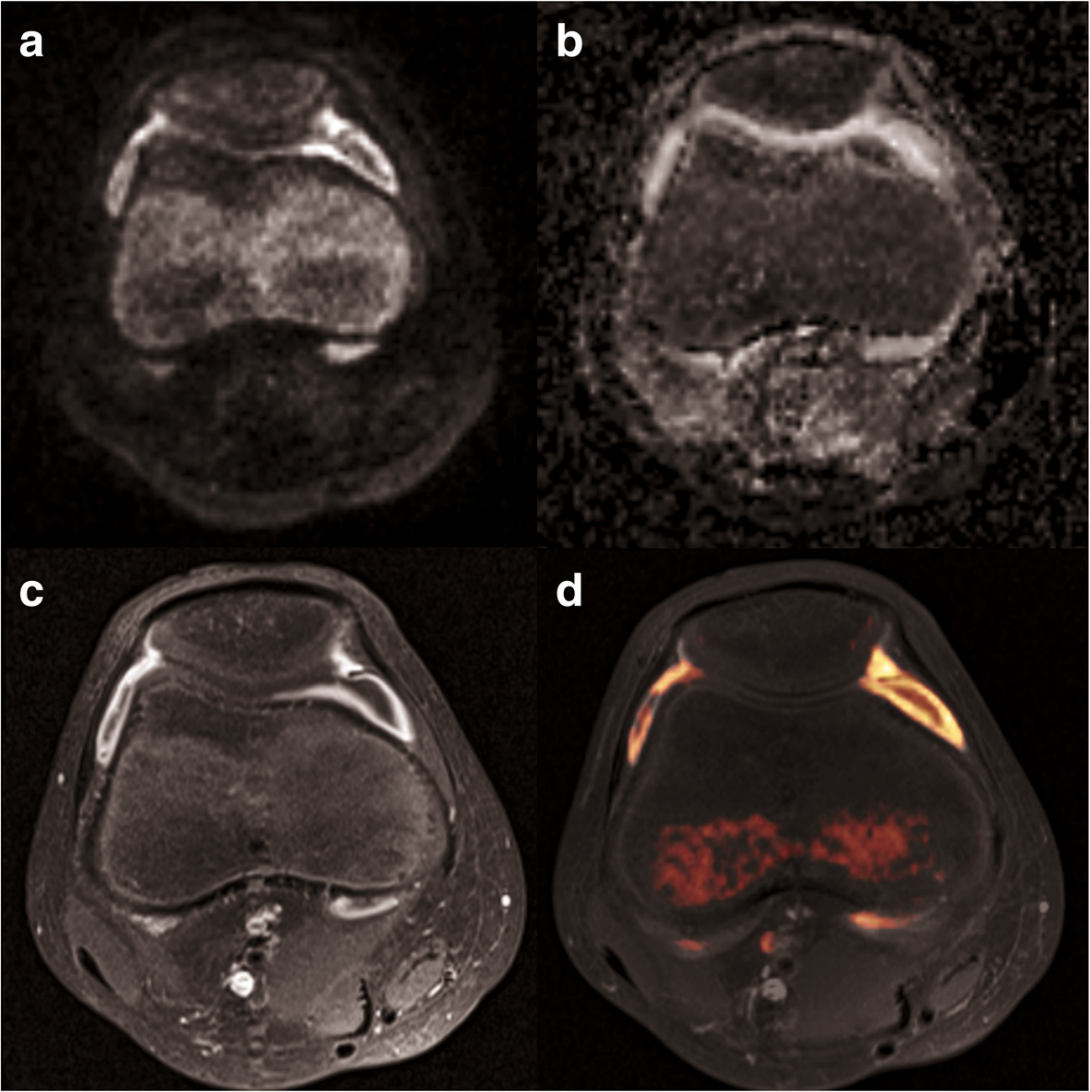
Twelve-year-old male JIA patient, persisting clinical symptoms with methotrexate treatment. DWI b = 800 s/mm^2^ (**a**), ADC map (**b**), contrast-enhanced MRI (**c**) and image fusion (**d**) of colorised overlay of DWI signal onto image (**c**) with 3D Fusion software (Siemens Medical) all show active synovitis and small amounts of joint effusion in the anterior and posterior joint space. Image acquisition with multi-shot read-out segmented DWI (RESOLVE, Siemens) is virtually free of artifacts

All image analysis was performed on a dedicated radiological workstation (Merlin Version 5.1., Phoenix PACS GmbH, Freiburg, Germany). A board-certified paediatric radiologist with 8 years of experience in paediatric extra-cranial DWI (1st reader), a senior resident radiologist trained in paediatric imaging (2nd reader) and a radiation oncologist with basic training in musculoskeletal MRI, reflecting three different levels of experience, independently completed all qualitative image analyses. Each reader, blinded to all clinical patient data and to the results of the other readers, assessed contrast-enhanced images and then diffusion-weighted images in separate reading sessions (Fig. 1). Each reader recorded the presence and the degree of synovitis on a modified 5-item Likert scale: 0 = non-diagnostic image quality; 1 = no signs of synovitis; 2 = minimal circumscript or linear signal increase, not qualifying for active synovitis; 3 = active synovitis present, defined as marked linear signal increase of the synovial layer with 1–2 mm in thickness; 4 = active synovitis plus circumscript or diffuse synovial thickening in excess of 2 mm. Categories 3 and 4 were considered to represent active synovitis/arthritis, while category 1 was thought to exclude the presence of active synovitis, based on MR imaging criteria. Category 2 indicated mild synovial irritation not qualifying for active synovitis. Along with each rating on synovitis, the respective subjective level of diagnostic confidence (LoC) was documented on a modified 3-item Likert scale: 1 = undecided, questionable, low confidence; 2 = intermediate, diagnosis established with some confidence; 3 = certain diagnosis, high confidence. The contrast-enhanced MRI scan was considered the diagnostic standard of comparison.

**Fig. 2:**
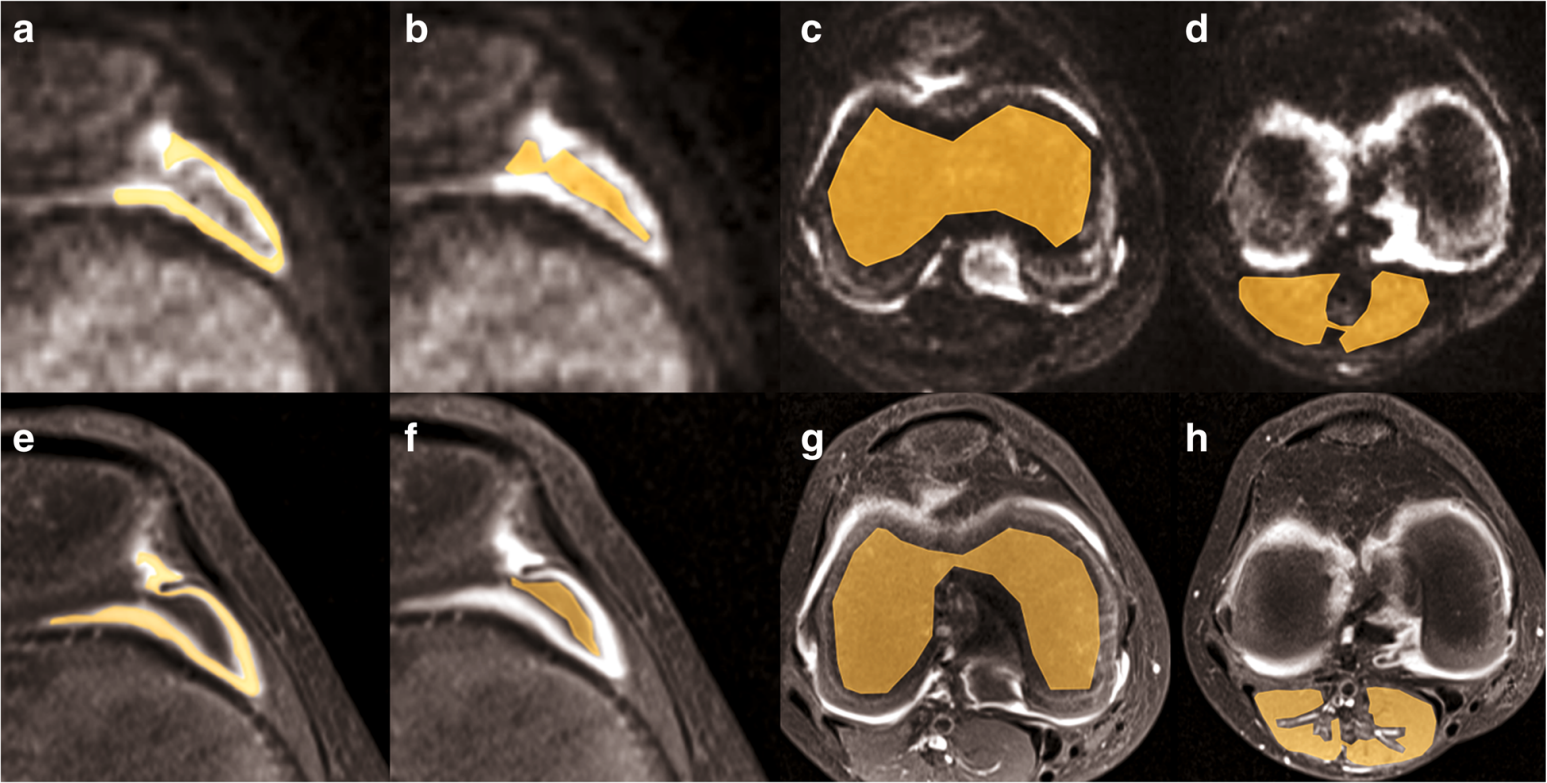
Same patient as in Fig. 1. Region of interest (ROI) placement using half-translucent polygonal ROIs on DWI b = 800 s/mm^2^ (**a**-**d**) and contrast-enhanced MRI (**e**-**h**) for manual segmentation of synovium (**a**, **e**), joint effusion (**b**, **f**), femoral epiphyseal bone marrow (**c**, **g**) and upper calf muscle (**d**, **h**)

The same experienced paediatric radiologist blinded to patient data and to the results of preceding qualitative image analysis also performed quantitative analysis using polygonal Regions of interest (ROIs) manually drawn on one representative cross-section of contrast-enhanced images, diffusion-weighted images (Fig. 2) and the corresponding ADC map. Signal intensities were measured for synovium, joint effusion, upper calf muscle, femoral epiphyseal bone marrow and popliteal lymph nodes on contrast-enhanced MRI, DWI at high b-values and on the ADC map. The apparent diffusion coefficient (ADC) was recorded as a quantitative measure of tissue diffusivity. We calculated signal intensity ratios for synovium vs. effusion, synovium vs. muscle etc., so as to quantify image contrast between different anatomical structures. Quantitative analysis was repeated by the same reader after one week for assessment of intra-observer variability.

### Statistical analysis

Normally distributed data is presented as mean ± standard deviation and range, data deviating from normal distribution as median with inter-quartile range. The Wilcoxon paired-sample test was employed to compare means of two related data sets deviating from normal distribution. Kappa statistics were calculated to assess intra-observer and inter-observer agreement. The coefficient of variation was computed to quantify intra-observer variation in quantitative data. All data analyses were performed with SPSS Version 21 for Windows (IBM SPSS Statistics for Windows, Armonk, NY, USA).

## Results

All MRI examinations were completed safely. All scans were consistently rated as diagnostic by all three readers.

### Contrast-enhanced MRI as diagnostic standard

**Fig. 3:**
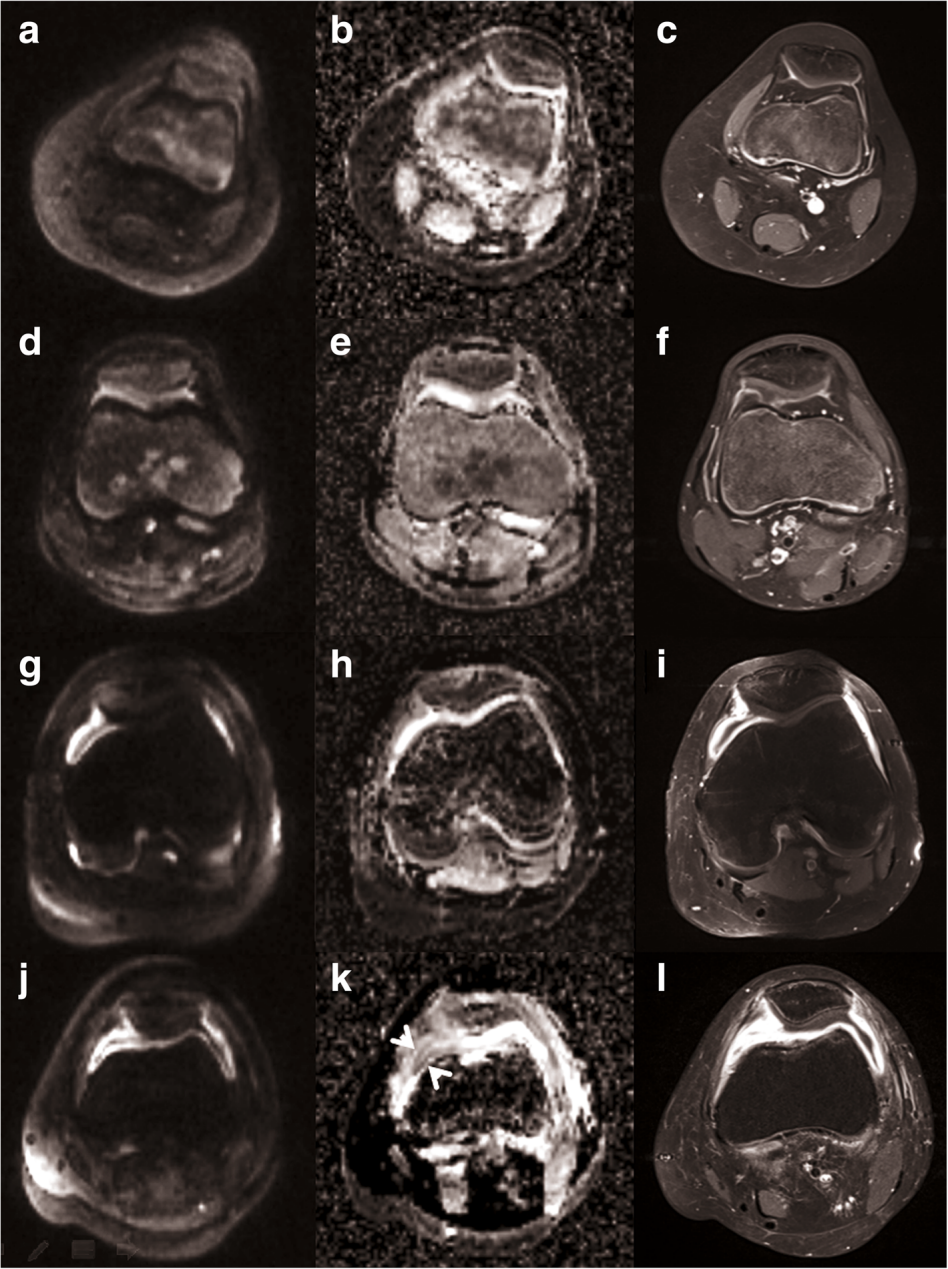
Corresponding transverse cross-section of DWI b = 800 s/mm^2^ (**a**, **d**, **g**, **j**), ADC map (**b**, **e**, **h**, **k**), contrast-enhanced T1w imaging (**c**, **f**, **i**, **l**) in four different patients, rated as not having synovitis (category 1, **a**-**c**), showing mild synovial irritation (category 2, **d**-**f**), active synovitis with joint effusion (category 3, **g**-**i**) and synovitis aggravated by synovial thickening (category 4, **j**-**l**) on contrast enhanced MRI as the diagnostic standard. The fourth patient (**j**-**l**) was rated as category 3 on DWI, since the synovial layer appears less prominent and since the ADC map nicely delineates synovium from joint effusion (arrow heads) and periarticular oedematous signal increase. Contrast-enhanced MRI may overestimate the extent of synovial proliferation in the presence of perisynovial inflammation and oedema in this patient

The reading completed by the 1st reader was considered the diagnostic standard of comparison. The 1st reader assigned patients to the categories of synovial signal as follows (Fig. 3): category 0 - no patients, as all scans were of sufficient diagnostic quality; category 1 - nine patients (28%) without any significant synovial contrast enhancement; category 2 - nine patients (28%) with minimal circumscript or linear synovial contrast uptake, not qualifying for active synovitis; category 3 - nine patients (28%) with marked synovial contrast uptake and mild synovial thickening not exceeding 2 mm; category 4 - five patients (16%) with strong synovial contrast uptake, marked synovial thickening and large joint effusion. Level of confidence (LoC) of the ratings by the 1st reader was high (LoC = 3) in 27 of 32 (84%) patients and medium (LoC = 2) in 5 (16%) patients. All patients in category 3 and 4 showed varying degrees of joint effusion. In contrast, none of the patients in category 1 and 2 had significant effusion. With regard to underlying pathology, none of the eight control patients without JIA had active synovitis (category 1 *n* = 6; category 2 *n* = 2). The 24 JIA patients were assigned to the categories of synovial signal as follows: categorry 1 - three patients with previously diagnosed JIA, all of them under on-going anti-inflammatory treatment; category 2 - six patients, among these three newly diagnosed JIA patients and three of six patients under anti-inflammatory treatment; category 3 - nine patients, four of these newly diagnosed with JIA, and seven patients undergoing inflammatory treatment including four patients with persisting arthritis under methotrexate; category 4 - five patients, including one newly diagnosed two-year-old without previous anti-inflammatory treatment. Based on these findings, the categories 1&2 and 3&4 were combined into a dichotomous variable, presenting 0 = no arthritis and 1 = active arthritis, for further analysis.

### Diagnosis of synovitis on DWI

Compared to the diagnostic standard of contrast-enhanced MRI, the reading of DWI completed by the 1st reader showed consistent ratings in the four categories of synovial signal in 26 of 32 cases (81%) with a κ = 0.74 (*p* < 0.001), indicating substantial agreement across all categories based on the criteria by Landis and Koch [15]. All disconcordances occured from “down-grading” on DWI, that is two cases of category 4 on contrast-enhanced MRI were rated as category 3 on DWI, and another 4 cases of category 2 on contrast-enhanced MRI were assessed as category 1 on DWI. Interestingly, the downgrades from category 4 to 3 all were high confidence (LoC = 3) ratings on DWI, while the downgrades from category 2 to 1 all occured with low confidence (LoC = 1) on DWI. There was full agreement between the ratings on contrast-enhanced MRI and on DWI with regard to the presence or absence of active arthritis. The overall diagnostic level of confidence was lower on DWI, compared to contrast-enhanced MRI, including 14 ratings with high, 8 ratings with intermediate and 11 ratings with low diagnostic confidence. All low-confidence ratings pertained to choosing between categories 1 and 2.

### Inter-observer agreement on contrast-enhanced imaging

Compared to the reference reading performed by the first observeron on contrast-enhanced MRI images, the ratings of the other observers showed moderate agreement. Kappa values across all categories were 0.50 (*p* < 0.001) for the 2nd reader and 0.48 (p < 0.001) for the 3rd observer. Agreement between 2nd and 3rd reader was 0.54 (p < 0.001). Comparing the ratings of the 2nd reader to the reference reading, there were one upgrade from category 1 to 2, six downgrades from category 2 to 1, three upgrades from category 3 to 4 and one downgrade from category 4 to 3. The 3rd reader passed higher ratings in seven patients (all upgrades from category 1 to 2) and lower ratings in five patients (*n* = 1 category 2 to 1; n = 1 category 3 to 2; *n* = 3 category 4 to 3). As compared to reference, one patient with active synovitis (category 3) was therefore misdiagnosed as not having synovitis. Deciding on the presence or absence of active arthritis, there was full agreement between the first and second reader (κ = 1) and disagreement in one case between the first and the third reader (κ = 0.94, *p* < 0.001) on contrast-enhanced MRI.

### Inter-observer agreement on DWI

Compared to the reference reading on contrast-enhanced MRI performed by the first reader, the DWI ratings of the other readers showed fair to moderate agreement. Kappa values across all categories were 0.50 (p < 0.001) for the 2nd reader and 0.39 (p < 0.001) for the 3rd reader. Agreement of DWI ratings for the 2nd reader vs. 3rd reader was still fair with κ = 0.25 (*p* < 0.002). Comparing the DWI ratings of the 2nd reader to reference, there were one upgrade from category 1 to 2, six downgrades from category 2 to 1, four upgrades from category 3 to 4 and one downgrade from category 4 to 3. The 3rd reader passed higher ratings in eight patients (*n* = 5 category 1 to 2; *n* = 1 category 2 to 3; *n* = 2 category 3 to 4) and lower ratings in six patients (n = 1 category 2 to 1; *n* = 4 category 4 to 3). Therefore, the 3rd reader considered one patient without active synovitis as having synovitis.

With regard to the presence or absence of active arthritis on DWI, we thus found full agreement for the second reader (κ = 1) and discrepancy in one case for the third reader (κ = 0.94, *p* < 0.001) on diffusion-weighted imaging, as compared to the diagnostic standard.

### Observer-dependent level of confidence

Diagnostic level of confidence (LoC) showed the following median values for confidence ratings: median LoC =3 on contrast-enhanced MRI and on DWI for the 1st reader, median LoC = 3 on contrast-enhanced MRI and median LoC = 2 on DWI for the 2nd reader and the 3rd reader. The proportion of high-confidence ratings (LoC = 3) was 84%, 81% and 72% on contrast-enhanced MRI and 44%, 32% and 0% on DWI for the 1st, 2nd and 3rd reader, respectively.

### Quantitative analysis

Mean ADC (unit: 10^−3^ mm^2^/s) of inflamed synovium (2.08 ± 0.24, range 1.64 to 2.42) was lower than mean ADC of joint effusion (2.85 ± 0.55, range 2.33 to 3.31), but higher than mean ADC of muscle (1.61 ± 0.29, range 1.36 to 1.88) and lymph nodes (1.11 ± 0.16, range 0.74 to 1.34). ADC of muscle tissue showed the smallest standard deviation and the smallest between-measurements coefficient of variation (3.0%) and was therefore chosen as standard internal reference for signal comparison. DWI showed high intrinsic signal for inflamed synovium and popliteal lymph nodes, compared to muscle signal. Median intensity ratios of synovium signal vs. muscle (2.7, interquartile range [IQR] 1.5) and of lymph nodes vs. muscle (2.8, IQR 1.8) were higher on DWI than on contrast-enhanced MRI (2.9, IQR 0.9, and 1.9, IQR 0.4, respectively) (both *p* < 0.001). In contrast, signal intensity ratio of synovium vs. joint effusion was significantly lower on DWI (1.5, IQR 0.5), compared to contrast-enhanced MRI (2.7, IQR 0.7) (p < 0.001). The coefficient of variation for two repeated measurements by the same reader ranged less than 10% for all measured tissues.

## Discussion

In paediatric non-oncological MRI, imaging of synovitis is probably the most common of only few good indications for i.v. gadolinium-containing contrast injection. Although there has been research on gadolinium-free synovial scanning, such as arterial spin-labelling MRI [16] or DWI [7–10], the clinical impact of these techniques remains limited to date for various reasons, including lengthy image acquisition and post-processing, limited spatial resolution and proneness to imaging artifacts, among others. Single-shot echoplanar diffusion-weighted imaging has been state-of-the art in stroke imaging since the early 1990s and also visualises synovitis in knee joints [7, 10]. In extra-cranial anatomical regions, however, such DWI scans are frequently degraded by the presence of artifacts, such as ghosting, distortion or the so-called “T2w shine-through” arising from joint effusion which spoil diagnostic visibility of the synovial layer in about one-third of patients [11].

**Fig. 4:**
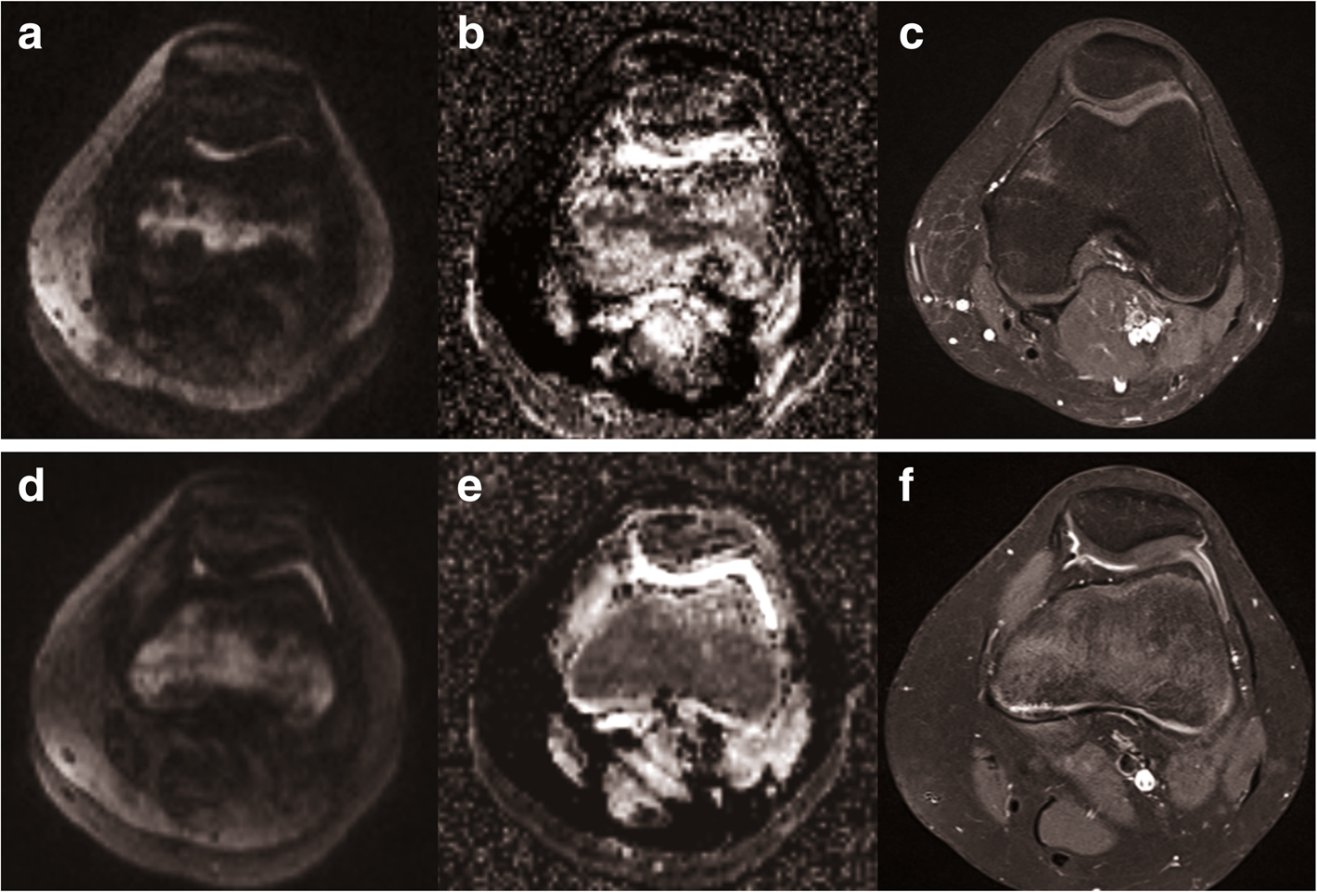
Diagnostic dilemma in a 14-year-old female control patient without synovitis (**a**-**c**) and in a 13-year-old female patient (**d**-**f**) newly diagnosed with JIA based on International League of Associations for Rheumatology (ILAR) criteria [14]. While the first patient does not show any synovial contrast enhancement (category 1, **c**), there is mild synovial enhancement and some synovial fluid discernable in the JIA patient (category 2, **f**). Both patients, however, display retropatellar and parapatellar high signal on DWI b = 800 s/mm^2^. Some signal increase as in (**a**) is frequently encountered in normal knee joints, possibly due to “T2w shine-through” of small amounts of physiological joint fluid, making diagnostic differentiation of synovial signal category 1 and 2 difficult, or impossible

Based on our study results, the recently developed multi-shot DWI sequence (e.g. RESOLVE, Siemens), as used in our study, reliably visualises inflamed synovium in patients with active arthritis of the knee joint and is superior to the standard single-shot DWI. Multi-shot DWI is scanned in several steps, or “segments”, and composes a qualitatively enhanced MR image with higher spatial resolution and less artifacts. In our study, voxel size was 1.5 × 1.5 × 3 mm^3^, as compared to 1.8 × 1.8 x (4 to 6) mm^3^ with single-shot DWI in earlier studies [7, 11]. We observed less “T2w shine-through” artifacts, although these artifacts still occur in some patients. Limited diagnostic accuracy was observed in knee joints with little synovial inflammation in the presence of slightly increased amounts of synovial fluid (Fig. 4), as commonly seen with mild synovial irritation in patients with trauma, over-exertion or residual arthritis under anti-inflammatory treatment. This limitation would be even more serious when imaging small joints, such as wrist, finger or temporomandibular joints, where T2w shine-through artifacts could more easily mimick synovial signal increase and cause false-positive ratings [8, 9].

Using multi-shot DWI, mean ADC of synovium and of joint effusion fell within the range previously reported from single-shot DWI scans [7] and from an 1 Tesla open-bore scanner [10], while ADC of upper calf muscle tissue was substantially higher than the 1.17 ± 0.12 × 10^−3^ mm^2^/s previously measured in skeletal muscle [17]. Generally, muscle tissue presents a good internal reference standard for ADC, as large and rather homogeneous tissue volumes can be measured with little partial volume effects and low between-measurement variation on the same scanner.

Qualitative evaluation of synovial signal was performed by three independent readers with different levels of expertise in musculoskeletal MRI. Diffusion-weighted imaging, as used in our study, counts among the technically advanced MRI acquisition methods. Spatial resolution is low, by comparison. And while DWI images contain a treasure of diagnostic information, the aesthetic effect of these images is not particularly striking. In our study, we were thus interested in knowing whether DWI of the knee joint is only for experts, or whether “normal” radiologists and radiologically trained non-radiologists can also work with such images. We thus chose three independent readers at respective levels of experience. Our results demonstrate that diagnostic accuracy indeed is a function of experience and also that the interpretation of diffusion-weighted images is generally more demanding, compared to contrast-enhanced MRI. Nevertheless, the crucial diagnostic decision of seeing or not seeing active arthritis was made with rather high accuracy and with low inter-observer variation. We are therefore convinced that, after some training, the new DWI technique presented in our study can readily be employed by radiologists in clinical routine imaging. The lower diagnostic confidence recorded for ratings on DWI is, in our view, directly related to the lower image contrast of inflamed synovium vs. effusion in combination with the still lower spatial resolution of DWI, as compared to contrast-enhanced MRI. In the absence of synovitis, there is no discernible DWI signal from the synovial membrane, which measures only a couple of cell layers in width. With increasing activity of synovitis, the synovial signal becomes visible and gradually more prominent. Eventually, synovial proliferation, as in chronic synovitis or pannus, produces strongly altered local diffusivity and shows most clearly. However, the presence of joint effusion, which usually coincides with synovitis at varying degrees, makes things more complicated. By the so-called “T2w shine-through” artifact, effusion can produce some signal very close to synovitis on DWI. If there is little or no synovitis in the presence of small amounts of joint effusion, be it arthritic or non-arthritic in origin, it is difficult or impossible to decide whether the observed signal originates from synovitis or from T2 signal effects of the joint effusion. This presently limits the usefulness of DWI in joints with little inflammatory activity. In patients with severe synovitis and large effusion, the synovium is usually prominent enough to be easily distinguished from effusion. Advanced DWI techniques, such as intravoxel incoherent motion (IVIM) imaging [18, 19] visualize both synovial diffusivity and synovial perfusion. These techniques are probably superior to multi-shot DWI under difficult scanning conditions and promise to overcome the technical difficulties arising from joint effusion [20].

The main methodological limitation of our study is the retrospective data analysis. Diagnostic accuracy of DWI in patients with arthritis should be evaluated in a prospective setting with contrast-enhanced imaging as the diagnostic standard.

In summary, imaging of synovitis with multi-shot DWI is feasible in a clinical routine setting and holds considerable clinical potential to increase patient safety and comfort, to streamline MRI scan protocols for arthritis patients and to reduce cost.

## Conclusion

Based on our study results, multi-shot DWI recommends itself as a non-invasive gadolinium-free MR scanning technique for imaging of synovitis. Using multi-shot echoplanar imaging with segmented-readout, DWI consistently produces images of good diagnostic quality and reliably visualises active synovitis in JIA patients with arthritis of the knee joint. “T2w shine-through” originating from joint effusion still limits diagnostic accuracy in patients with mild synovial irritation. The diagnostic utility of multi-shot DWI for imaging small joints should be investigated in future prospective studies.

## Abbreviations

ADC: Apparent diffusion coefficient
DWI: Diffusion-weighted imaging
IQR: Inter-quartile range
JIA: Juvenile idiopathic arthritis
LoC: Level of confidence
ROI: Region of interest
